# Removing 65 Years of Approximation in Rotating Ring Disk Electrode Theory with Physics-Informed Neural Networks

**DOI:** 10.1021/acs.jpclett.4c01258

**Published:** 2024-06-10

**Authors:** Haotian Chen, Bedřich Smetana, Vlastimil Novák, Yuanmin Zhang, Stanislav V. Sokolov, Enno Kätelhön, Zhiyao Luo, Mingcheng Zhu, Richard G. Compton

**Affiliations:** † Department of Chemistry, Physical and Theoretical Chemistry Laboratory, University of Oxford, South Parks Road, Oxford OX1 3QZ, Great Britain; ‡ Department of Chemistry and Physico-chemical processes, Faculty of Materials Science and Technology, VSB - Technical University of Ostrava, 17. listopadu 2172/15, 708 00 Ostrava-Poruba, Czech Republic; § St John’s College, University of Oxford, St Giles’, Oxford OX1 3JP, Great Britain; ∥ Independent Researcher, Offenbach am Main 63067, Germany; ⊥ Department of Engineering Science, University of Oxford, Parks Road, Oxford OX1 3PJ, United Kingdom; # Department of Computing, Imperial College London, Exhibition Road, London SW7 2AZ, United Kingdom

## Abstract

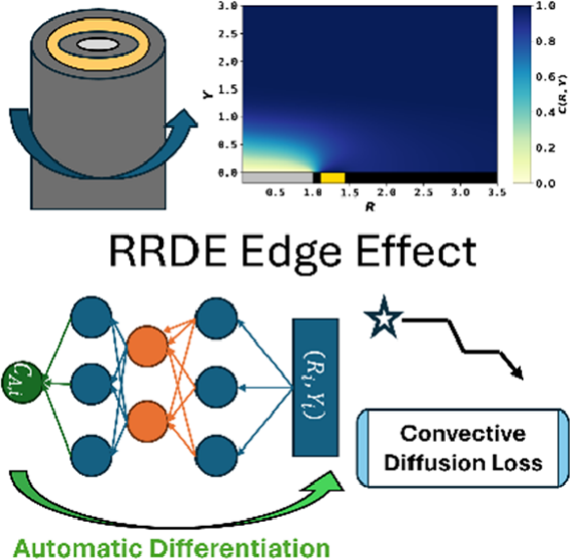

The rotating Ring Disk Electrode (RRDE), since its introduction in 1959 by Frumkin and Nekrasov, has become indispensable with diverse applications in electrochemistry, catalysis, and material science. The collection efficiency () is an important parameter extracted from the ring and disk currents of the RRDE, providing valuable information about reaction mechanism, kinetics, and pathways. The theoretical prediction of  is a challenging task: requiring solution of the complete convective diffusion mass transport equation with complex velocity profiles. Previous efforts, including by Albery and Bruckenstein who developed the most widely used analytical equations, heavily relied on approximations by removing radial diffusion and using approximate velocity profiles. 65 years after the introduction of RRDE, we employ a physics-informed neural network to solve the complete convective diffusion mass transport equation, to reveal the formerly neglected edge effects and velocity corrections on , and to provide a guideline where conventional approximation is applicable.

The Rotating Ring Disk Electrode (RRDE) is a widely used electrochemical measurement tool introduced by Frumkin and Nekrasov in 1959.^1^ The RRDE emerged following the earlier development of the Rotating Disk Electrode (RDE),^2,3^ which allowed the precise control of the electrode’s rotation speed, so that the rotation enhances mass transport to and from the electrode surface in a well-defined manner, permitting the separation of mass transport and electrode kinetics effects in steady-state voltammetry. As illustrated in Figure 1, the RRDE consists of a central disk electrode, a concentric ring electrode, and an electrochemically inactive annular separator between the two electrodes. The laminar flow associated with the electrode rotation (see Figure 1a) is such that fresh solution is drawn to the disk electrode, where it undergoes electrolysis with the reaction intermediates and products swept away from the surface by the flow. As these species spin out radially, they encounter the ring electrode, typically held at a different potential to that applied to the disk. In this way, by controlling and varying the ring potential, it is possible to record a voltammogram reflecting the chemical identity of the species reaching the ring electrode. Thus, the ring electrode is typically used to detect and quantify reaction products formed during the electrochemical processes occurring at the disk electrode, including the Oxygen Evolution Reaction and the Oxygen Reduction Reaction (OER/ORR).^4^ The application to the ORR was reported in the original paper by Frumkin and Nekrasov^1^ where the reduction of oxygen, O_2_, on a Au/Hg amalgam disk was shown to result in the formation of hydrogen peroxide (the 2 electron reduction product) at low reduction potentials but water (the 4 electron product) at more negative disk potentials. In general, the “collection” of reaction intermediates and products at the ring electrode provides valuable information about the reaction mechanisms, rates, and pathways. Today, the RRDE has become an indispensable tool for fuel cells, electrocatalysis, and electroanalytical research. Notable applications include the direct determination of the fraction of peroxide production during ORR on supported catalysts,^5^ evaluating OER/ORR activities,^6,7^ in situ measurements of interfacial pH,^8^ and probing the lithium–sulfur redox reactions.^9^

**Figure 1:**
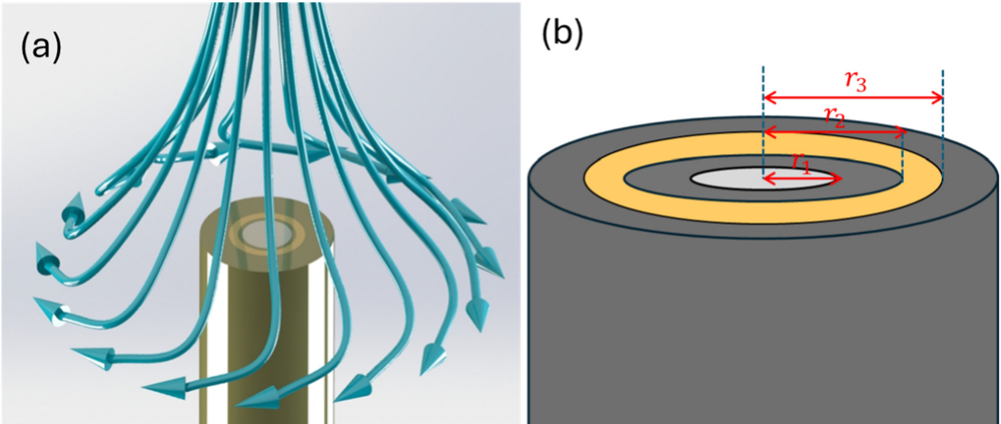
(a) Schematic illustration for the geometry of the RRDE and the flow patterns created by the RRDE. (b) Definition of *r*_1_, the disk radius, *r*_2_, the inner radius of the ring, and *r*_3_, the outer radius of the ring.

The full partial differential equation that describes the mass transport for chemically stable species at the (R)RDE is
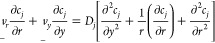
1where *c*_*j*_ and *D*_*j*_ are, respectively, the concentration and diffusion coefficient of the arbitrary species *j* and it is assumed that migration plays no role.  and  are the radial and normal components of the fluid velocity created by the rotation of the RRDE,^10,11^ as follows:
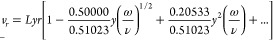
2
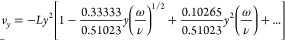
3where ω is the rotation speed (rad/s), ν is the kinematic viscosity, and *L* is the convection constant for ν as defined in Table 1.

**Table 1:** Definition of the Dimensional Parameters

Variable(s)	Symbol(s)	Definition(s)	Unit
Radius of disk, inner radius of ring, outer radius of disk	*r*_1_, *r*_2_, *r*_3_		*m*
Concentration and bulk concentration of species *j*	*c*_*j*_, *c*_j_^*^		*mol*/*m*^3^
Diffusion coefficient of species *j*	*D*_*j*_		*m*^2^/*s*
Applied potential and formal potential	*E*, *E*_f_^0^		*V*
Frequency of rotation	*f*		*Hz*
Kinematic viscosity	ν (Greek letter)		*m*^2^/*s*
Scan rate	***v*** (English letter, bold)		*V*/*s*
Fluid velocity in (x, y, z) direction	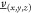		*m*/*s*
Approximate fluid velocity in y direction	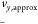	–*Ly*^2^	*m*/*s*
Approximate Fluid Velocity in r direction	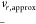	*Lyr*	*m*/*s*
Rotation speed	ω	2*πf*	*rad*/*s*
Reynolds number	Re	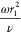	Dimensionless
Schmidt number	Sc	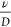	Dimensionless
Convection Constant	*L*	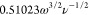	*m*^–1^*s*^–1^
Diffusion layer thickness	*δ*_D_	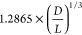	*m*
Hydrodynamic layer thickness	*x*_H_	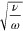	*m*

An important parameter for the RRDE is the collection efficiency, *N*, where 0 < *N* < 1 is the fraction of the material generated at the disk electrode, which is detected at the ring electrode if it is assumed that the disk reaction is a simple *n* electron transfer:
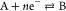
4with the reverse process
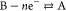
5at the ring and where it is further presumed that both processes are under mass transport control. Note that *N* < 1 since some B is lost to bulk solution. Thus, it can be expected that *N* is larger for RRDEs with smaller gaps between the ring and disk and larger rings. Due to the complexity of the 2-D convective diffusion equation, historical efforts to solve eq 1 and obtain the collection efficiency relied on several simplifying physical approximations as discussed below. The efforts to calculate *N* analytically started from the work of Ivanov and Levich in 1959 using a contour integration method, which gave a good (but far from perfect) agreement with experiments made on the reduction of benzoquinone in aqueous solution.^4^ The analytical expression for the theoretical collection efficiency developed by Ivanov and Levich is
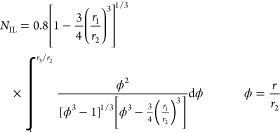
6where *r*_1_, *r*_2_, and *r*_3_ are the radius of the disk, inner radius of the ring, and outer radius of the ring, respectively (see Figure 1b).

*N*_IL_ is the first reported approximate analytical expression of collection efficiency for RRDE. However, due to the difficulty of solving the integral—numerical integration was done “by hand” with facilitating approximations and not computationally at the time—it suffered from errors as high as 15% due to the adoption of approximation to facilitate the numerical evaluation, which was less than perfect in some applications for the required level of electrochemical accuracy.^12^ Subsequently, and apparently inspired by a visit from the USA to the inventors of the RRDE in Moscow, Bruckenstein solved a key integral analytically^13^ and was able to generate the first closed form equation for the collection efficiency:
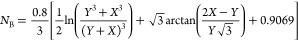
7where:
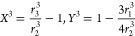
8

Subsequently Bruckenstein and Albery further refined the mathematics. They published the first “exact” solution^12^ based fully on the Ivanov/Levich physical model with the same assumptions but with more developed mathematics:
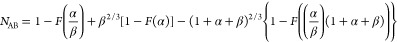
9where:
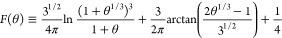
10
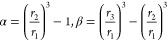
11

Although *N*_AB_ is often suggested to provide an “exact” solution for the collection efficiency at RRDE, it is an approximation; the mass transport equation underlying *N*_AB_ assumed the disk was uniformly accessible with no edge effects and ignored the radial diffusion:
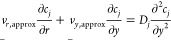
12where the convective flows were further approximated as
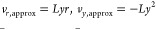
13

The neglect of edge effects in predicting the currents at an RDE are known to give rise to errors of the order of 1% in rotating disks of typical practical geometries^14,15^ while the truncation of eq 3 to one term leads to rather larger errors especially for solutes of high diffusion coefficients.^14,15^ These two effects operate in opposing directions for the RDE but do not cancel. However, in the context of the RRDE, Albery and Bruckenstein^12^ reported theoretically calculated and experimentally measured collection efficiency data to a much higher level of agreement than what might be expected on the basis of the same underlying assumptions made in the context of the RDE. Disappointingly details of the experiments made by Albery and Bruckenstein were not reported beyond the electrochemical systems employed, nor did the two individuals performing measurements appear as coauthors of the paper (but are thanked in an acknowledgment).

Despite the approximate nature of *N*_AB_, it is still perceived as the “gold standard” of the theoretical maximum collection efficiency as of today.^12,16−19^*N*_AB_ suggests that collection efficiency is only a function of the relative geometry of ring and disk and unaffected by the absolute size of electrodes and rotational speed. Nevertheless, in light of the results and modeling for the RDE discussed above, *N*_AB_ may likely not hold true in some experimental scenarios.

In this paper, we introduce the application of a physics-informed neural network (PINN) to lift the 65-year-old approximations to eq 1 to reveal the influence, if any, of the nonuniform accessibility to the disk electrode, of radial diffusion, and of truncation of the two-dimensional velocity profiles (eqs 2 and 3) to provide a more physically realistic collection efficiency by explicitly solving the fluxes at the disk and the ring electrode simultaneously. We especially seek to define the electrode size and experimental conditions, notably rotation speed, where *N*_AB_ might be applied with confidence.

The recent introduction of PINN,^20^ as a novel discretization-free partial differential equation (PDE) solver, has proven effective and accurate, to solve complicated PDEs in various domains, from modeling and reconstructing fluid mechanics flow fields,^21,22^ to material fatigue prediction and solid mechanics,^23,24^ and to blood pressure and hemodynamics estimation in healthcare.^25,26^ In the field of electrochemistry, PINN has re-educated hydrodynamic electrochemistry simulation in areas ranging from single and double microband channel electrodes to the rotating disk electrode with analytical levels of accuracy.^15,27^ In 2024, PINN is no longer at its infancy, or is complementary to traditional finite difference and finite element methods.^28^ The Electrochemistry-Informed Neural Netwok (ECINN) embedded electrochemical kinetic laws with mass transport equations, achieving simultaneous discovery of electrochemical rate constants, transfer coefficients, and diffusion coefficients.^29^ PINN stands ready to solve electrochemical problems for the community, offering freedom from previously essential approximations both physical and mathematical. All simulation programmers used below are available on 10.5281/zenodo.11396577 with neural network weights for users’ convenience.

In the following, the electrochemical reaction taking place at the disk electrode is assumed to be a simple one-electron reduction (n = 1) as A + *e*^–^ ⇄ B, where A and B are stable solution-phase species, with a bulk concentration of A of *c*_A_^*^ and a bulk concentration of B of zero. Transport-controlled reoxidation of B to A is assumed to take place on the ring electrode. The diffusion coefficients of the two species are assumed equal so that only A is modeled and *c*_A_ + *c*_B_ = *c*_A_^*^. The analysis assumes a sufficient supporting electrolyte such that the mass transport is dominated by diffusion and forced convection.

Using the steady-state mass transport equation and the velocity profiles from eqs 1–3, the boundary conditions representing the mass transport controlled limited currents are
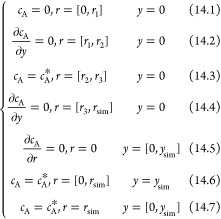
14where eqs 14.1 to 14.4 are the boundary conditions at the disk, the insulating gap between the ring and the disk, the disk, and the insulating shroud outside of the ring, respectively. Equation 14.5 is the boundary condition for the axis of rotation, and eqs 14.6 & 14.7 are the outer boundary of simulation where the bulk is unperturbed. The current at the disk electrode is calculated as
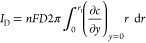
15

The current of the ring electrode is
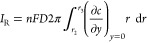
16

Simulations of RRDE were carried out in dimensionless space for multiple advantages. One of the most important advantages is avoiding the exploding/diminishing gradient problem by scaling the magnitude of variables, while reducing numerical instabilities and inaccuracies. In addition, dimensionless variables increased the generalization of the model to facilitate comparison in similar scenarios. Furthermore, the dimensionless variables were defined in Table 2, which were also widely adopted so that consistency across different models was ensured.
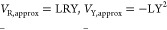


**Table 2:** Definition of Dimensionless Parameters^a^

Parameter	Dimensionless Form
Concentration	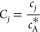
Diffusion coefficient	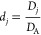
Spatial coordinate in y direction for 1-D simulation	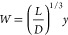
Spatial coordinate in y direction for 2-D simulation	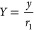
Spatial coordinates in r direction for 2-D simulation	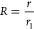
Approximate Fluid Velocity in *R* direction	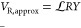
Fluid Velocity in *Y* direction under the Levich approximation	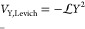
Time	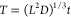
Dimensionless hydrodynamic constant	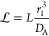
Potential	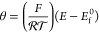
Flux at the disk electrode	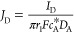
Flux at the ring electrode	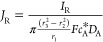

a *F*, , and  are the Faraday constant, the gas constant, and temperature, respectively. *E* and *E*_f_^0^ are the potential and formal potential, respectively.

The dimensionless form of the mass transport equation is
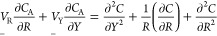
17where  and  are defined as
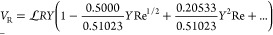
18
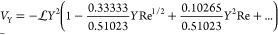
19

The approximate dimensionless velocity profiles, as used in the work of Ivanov and Levich and of Bruckenstein and Albery, were:
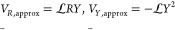
20

This section seeks to introduce how a PINN solves the above-formulated problem and includes a discussion of multiple sets of collocation domains, loss functions, and optimization methods.

PINN simulation of the steady-state RRDE problem seeks to solve the concentration *C*_A_(*R*, *Y*) as a function of dimensionless coordinates *R* and *Y* in the simulation domain Ω_R_ × Ω_Y_. The governing 2-D equation is challenging to solve: as , the exploding gradient problem will collapse the neural network when solving the concentration near *R* = 0. The “divide-and-conquer” strategy was adopted for the RRDE. Assuming a small area at *R* ∈ [0, *R*_0_] was uniformly accessible and unaffected by the edge effect, the concentration profile was approximated with an analytical expression as , where Γ is the regularized lower incomplete gamma function. *R*_0_ was a hyperparameter, and *R*_0_ = 0.05 was used for all simulations. As evidenced by convergence tests reported in the Hyperparameter Sensitivity section in the Supporting Information, *R*_0_ is small enough so that the electrode area inside *R* = *R*_0_ is unperturbed by any edge effect. The boundary conditions for PINN are
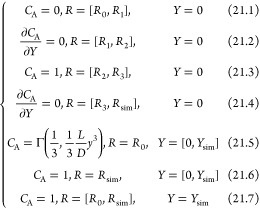
21where *R*_sim_ and *Y*_sim_ are the outer boundaries of simulation estimated from the diffusion layer thickness, *δ*_D_ and hydrodynamic layer thickness, *x*_H_ and defined below:
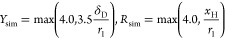
22where *δ*_D_ and *x*_H_ are defined in Table 1.  is directly provided by the neural network using automatic differentiation (AD).

In contrast to conventional data-driven neural networks, which make predictions by seeking correlation between known concentrations and their coordinates, PINN was trained by embedding PDEs and other physical constraints into their loss functions to solve them by minimizing the loss values on a large set of randomly distributed collocation points in the simulation domain. Figure 2a illustrates the structure of PINN and the collocation points used to solve the RRDE problem. For example, to enforce the mass transport equation,  collocation points were generated on the 2-D simulation domain using a uniform random distribution as  to ask a fully connected neural network to predict the concentrations  on these coordinates. An additional set of collocation points were placed near the electrodes to better resolve the mass transport region where electrochemical reaction happens. The predictions were then passed to the loss functions to calculate the errors, which were then minimized by the optimizer. The velocity field () described by eqs 18 & 19 is presented Figure 2b. and  were passed to the neural network as terms in the mean square error (MSE) to enforce the governing mass transport equation:
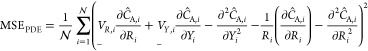
23

**Figure 2:**
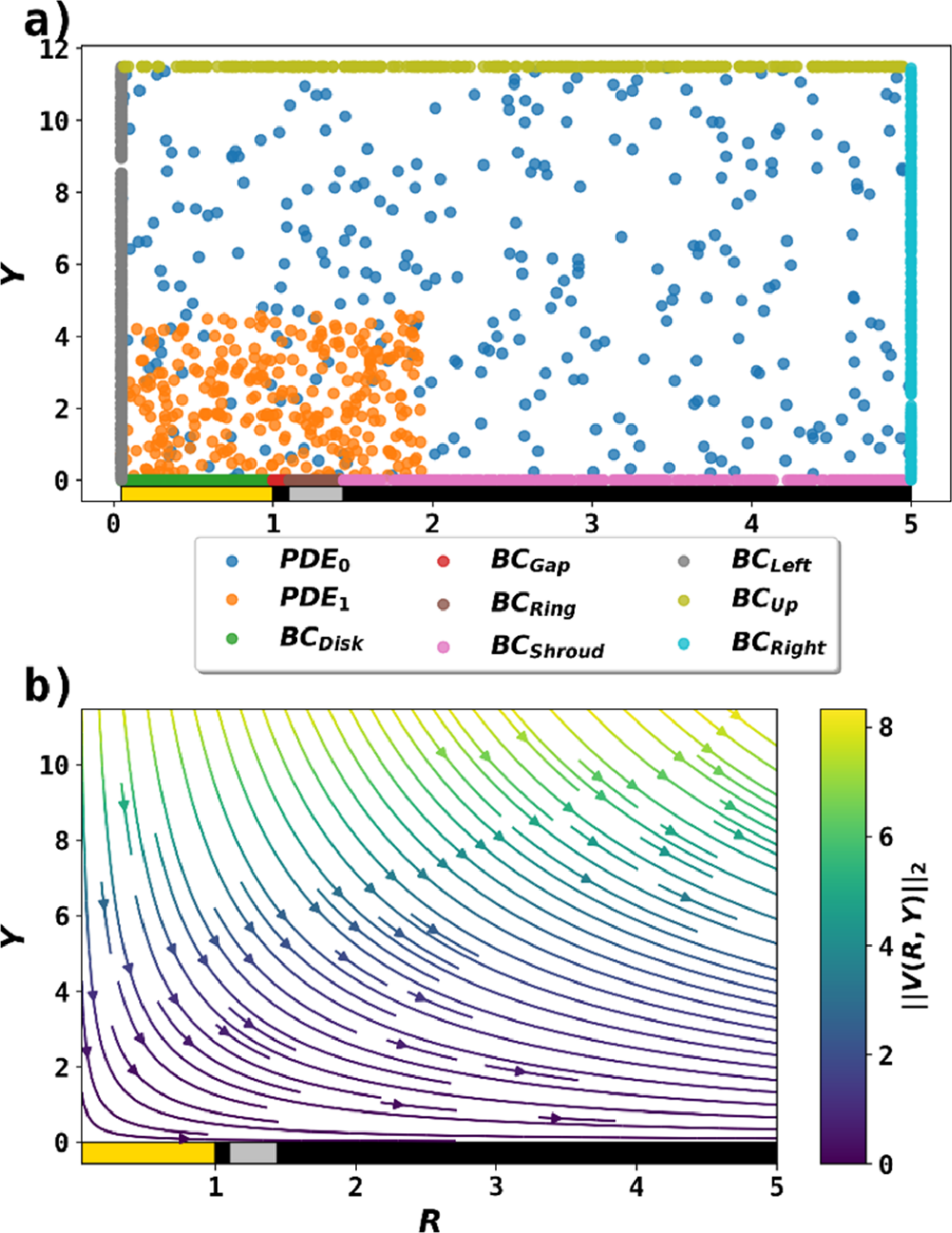
(a) A batch of collocation points containing 9 sets for PDE and boundary conditions. PDE0 enforces the convective-mass transport equation in the entire simulation domain, and PDE1 focuses on the domain near the electrodes for better accuracy. The other 7 sets enforce the boundary conditions. (b) The convective-diffusion mass transport velocity field calculated using eqs 17–19 with two correction terms when *r*_1_ = 10 μm, *r*_2_ = 11 μm, and *r*_3_ = 14.4 μm with rotation at 5 Hz. The gold and silver rectangles represent the disk and the ring electrode, respectively. The black rectangles are insulating material.

Similarly, the Dirichlet boundary conditions at the disk and ring electrodes were enforced with the following MSE loss functions.
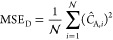
24
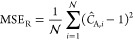
25

The no-flux boundary conditions (a Neumann boundary condition) on the gap and between the two electrodes and the shroud were similarly calculated using
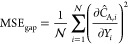
26
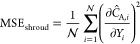
27

The overall loss () was the sum of individual losses enforcing the governing PDE and boundary conditions in eq 21:
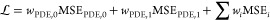
28where *w* is the weight of each MSE function to balance the magnitude of errors. During training, setting all weights to 1 was sufficient to achieve satisfactory accuracy. The PINN had five hidden layers with 128, 64, 64, 64, and 128 neurons in each layer. The activation function of all hidden layers was a hyperbolic tangent (tanh) as tanh was smoothly differentiable. The optimizer of PINN was Adam^30^ (learning rate = 10^–3^) and a learning rate scheduler decreased the learning rate by 2% for every epoch after 50 epochs. During simulations, the network was trained for 300 epochs and  was 8 million.

After training, a concentration profile is obtained, and the flux to the electrode surface is directly provided by neural networks as
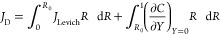
29
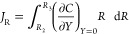
30

The collection efficiency is then calculated as
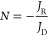
31

First, we report PINN simulation of the full convective diffusion mass transport for steady-state fluxes of a RRDE as a function of the square root of rotational speed in dimensionless form . To facilitate comparison with the analytical expression derived without Schmidt number (Sc) correction, the velocity profiles initially had no correction terms (eq 20). The influence of radial diffusion and the edge effect was evaluated, and the resulting collection efficiencies were compared with analytical expressions including *N*_AB_ and *N*_IL_.

The results are first discussed in dimensionless form (with increasing  and then discussed in a dimensional case (by proportionally increasing radii) to facilitate experimental understanding of this work. To convert between dimensional and dimensionless systems, the following variables were assumed, unless otherwise stated: *f* = 5 Hz, *D* = 10^–9^ m^2^ s^–1^, ν = 10^–6^ m^2^ s^–1^ at 298 K. Sc was thus fixed at 1 × 10^3^. The geometry of the RRDE was always fixed at *R*_1_ = 1, *R*_2_ = 1.1, and *R*_3_ = 1.44, so that the relative geometries are  and .

Second, PINN was further progressed by additional velocity correction terms (eqs 18 & 19) onto the governing mass transport equation to allow a more accurate solution of convective diffusion mass transport. Due to the complexity of the full equation with Schmidt number correction terms, numerical simulations have not previously been performed before to the best of the authors’ knowledge.

Lastly, the PINN simulation results were transformed to guide experimentalists in situations where radial diffusion and/or edge effects or the approximations in eq 20 are not negligible, and hence conventional analytical expressions are not applicable.

PINN allows solution of the convective-diffusion mass transport equation governing the RRDE to obtain fluxes on the disk and ring electrodes, respectively, for collection efficiency calculations. In addition, PINN can incorporate radial diffusion in eq 17 to reveal the edge effect on the electrodes, which is expected to be more significant with smaller electrodes. The approximate velocity profiles (eq 20) with no Schmidt number corrections were adopted to allow for comparison with conventional methods. To investigate the edge effect and the failure of conventional approximation, PINN was adopted to explicitly solve for the complete convective diffusion equation with radial diffusion by increasing  from 1.12 to 16.8. The approximate velocity profile (eq 20) was used in this part of the work.

Figure 3a shows the PINN solution for the concentration of A resulting from solution of the full convective diffusion equation when *r*_1_ = 30 μm at steady state, showing explicitly the depletion and regeneration of analyte on the disk and ring electrode, respectively, along with a nonuniform concentration gradient on the disk electrode. Note that the disk radius is selected to accentuate the effects of interest; as discussed below, electrodes in experimental practice have substantially larger radii. The diffusion/hydrodynamic layer thickness narrows from the center of the disk electrode to its edge, suggesting an increasing element of convergent diffusion to the electrode. Figure 3b illustrates a similar PINN simulation without radial diffusion, showing a uniformly accessible disk electrode and a thicker diffusion layer than if radial diffusion was imposed, as shown in Figure 3a. Figure 3c compares the flux densities for the two cases. First, the flux densities with radial diffusion are significantly larger in magnitude than flux densities without. Second, the large spike in flux density for the blue trace near *R* = 1 suggested strong radial diffusion contribution at the edge of disk electrode. Similarly, the sharp and negative flux density near *R* = 1.1 indicates a strong edge effect. Lastly, the small “hump” on the ring electrode around *R* = 1.3 indicates radial diffusion from the bulk back to the ring electrode, which, again and obviously, is not seen if radial diffusion is neglected.

**Figure 3:**
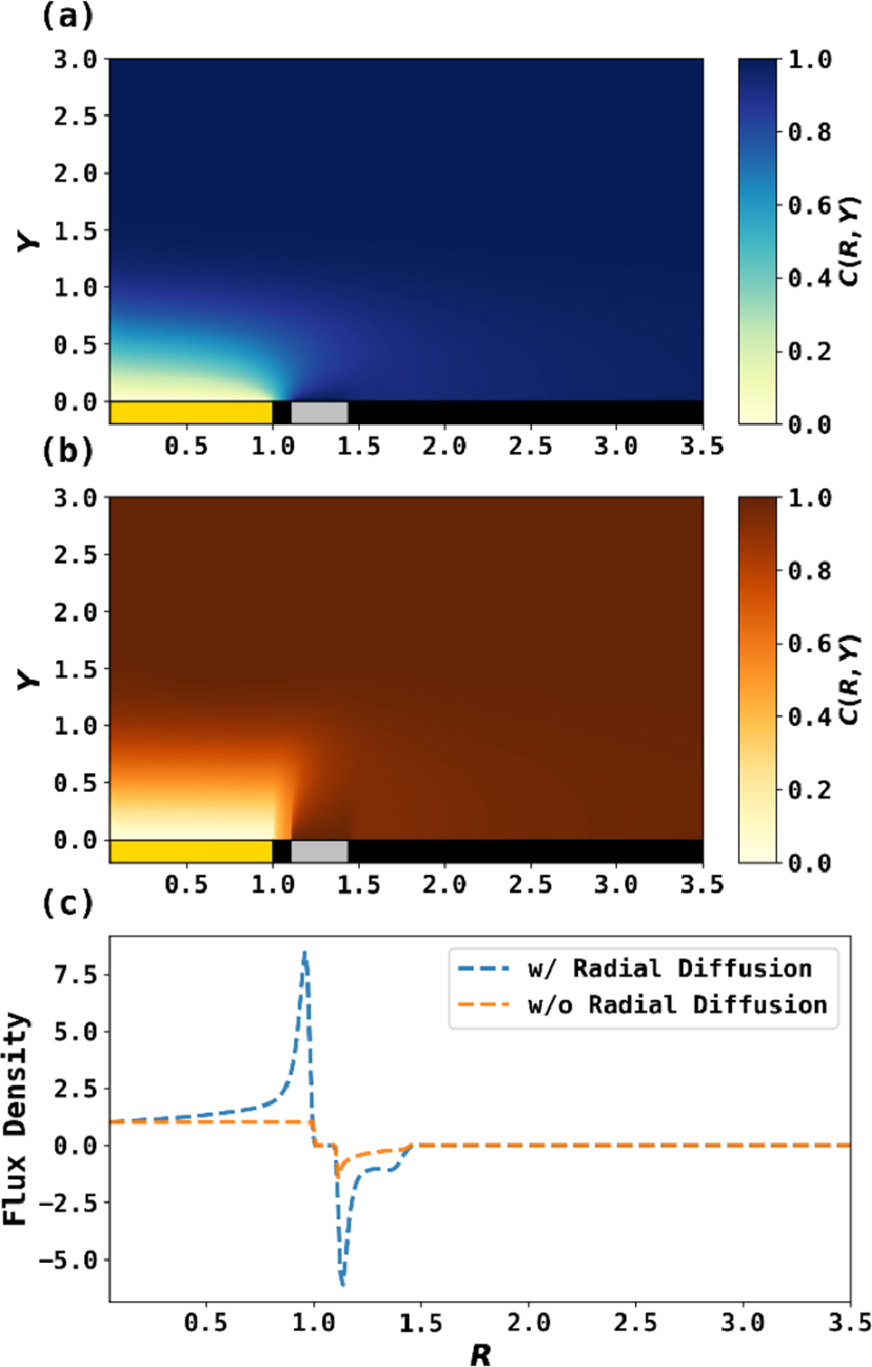
PINN simulation of a RRDE when  and without any Schmidt number correction. (a, b) The concentration profiles at steady state solved with radial diffusion (a) and without radial diffusion (b). (c) Comparison of the flux density at an RRDE with/without radial diffusion.

From Figure 3c, the fluxes on the disk and ring electrode with radial diffusion were *J*_D_ = 1.20 and *J*_R_ = −0.741, and thus a collection efficiency at *N* = 61.6%. The fluxes without radial diffusion predicted by PINN were *J*_D_ = 0.521 and *J*_R_ = −0.205, and thus *N* = 39.3%. The flux on the disk electrode in the absence of radial diffusion agreed well with the Levich equation as *J*_D, Levich_ = 0.522. The collection efficiency agreed well with Albery and Bruckenstein as *N*_AB_ = 40.0%, confirming the accuracy of the PINN method. This case study unveiled the non-negligible contribution of the radial diffusion to enhance the disk flux by 130% and the ring flux by 261%, such that the collection efficiency increased from 39.3% to 61.6%. The collection efficiency predicted by conventional analytical equations was *N*_AB_ = 40.0% or *N*_IL_ = 44.5%, which for the geometries studied underestimates the collection efficiency, reflecting the assumption of a uniformly accessible disk electrode and neglection of radial diffusion.

Figure 4 summarizes the PINN solution to the convective-diffusion equation with and without radial diffusion as the square root of rotational speed in dimensionless form  increased from 1.12 to 16.8. Figure 4a illustrates the ring and disk fluxes with and without radial diffusion. With radial diffusion, the ring and disk fluxes at lower  were both higher than the fluxes without radial diffusion. At higher , the contribution of radial diffusion diminished as convective mass transport dominated, and inclusion of radial diffusion has minimal effect on the predicted fluxes. Figure 4b systematically compared disk and ring fluxes with and without radial diffusion, with two major findings. First, the radial diffusion was more significant for the ring fluxes (light green curve) than for the disk fluxes (dark green curve). For example, fixing at 1.12, the ring flux was 500% higher with radial diffusion, while the disk flux was 220% higher. Second, radial diffusion was significant (>3%) when , such that the conventional solution to the RRDE by neglecting radial diffusion was no longer applicable in this region.

**Figure 4:**
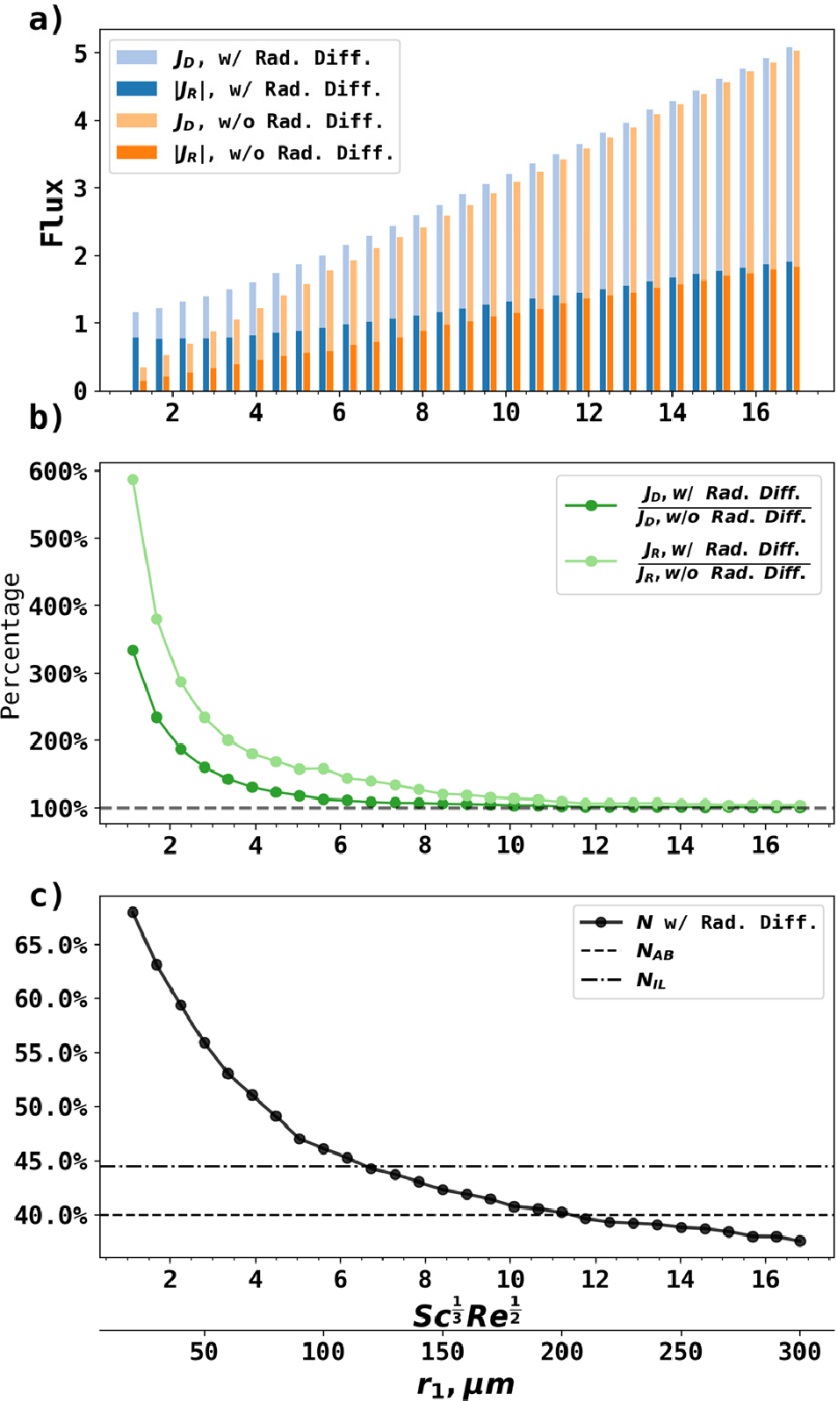
PINN solution to the RRDE as a function of increasing  with and without radial diffusion. No Schmidt correction was adopted for both cases. (a) The disk flux (*J*_D_), the absolute value of ring flux (|*J*_R_|) with and without radial diffusion. (b) The ratios of disk or ring fluxes with and without radial diffusion. (c) The collection efficiency *N* predicted by PINN with consideration to radial diffusion, *N*_AB_ and *N*_IL_. The secondary *x*-axis converts  to a function of *r*_1_ from 20 to 300 μm.

Figure 4c shows the collection efficiencies predicted by PINN by solving the full convective-diffusion equation with radial diffusion with no Schmidt number corrections. As the square root of rotational speed in dimensionless form  increased from 1.12 to 16.8, the collection efficiency calculated by PINN with radial diffusion decreased from ∼67% to ∼38%, and approaching *N*_AB_ with increasing . *N*_IL_, as an early predecessor of *N*_AB_, failed to capture the collection efficiency analytically. Note that *N*_AB_ is predicted to depend only on the relative geometry of RRDE and highlights the importance of taking the full equation into computation. PINN predicted that radial diffusion led to a slight decrease in collection efficiency as compared to *N*_AB_, which was attributed to the extra disk fluxes caused by the ring product back diffusion to the disk. The effect of back diffusion was neglected when deriving *N*_AB_. This case exemplifies the contributions of PINN to simulation of RRDE: to solve the more complicated convective diffusion mass transport equation explicitly and accurately without discretization. Figure 4 thus illustrates conditions where *N*_AB_ is appropriate.

To facilitate understanding with experiments, the dimensionless parameter  was set to 1.12 to 16.8, correspondeing to *r*_1_ = 20 to 300 μm when assuming a rotational frequency of 5 Hz and keeping the relative geometry of electrode at  and . These disk radii are typically lower than those used in practical RRDEs and larger than those of the microelectrodes typically used in stationary voltammetry. These geometries analyzed were selected to identify the physical effects before considering larger electrodes.

The effect of the Schmidt number correction on the velocity profile is discussed in the next section.

The conventional method for RRDE simulation typically ignores radial diffusion and utilizes the approximate velocity profile (eqs 12 & 13). In this section, the full convective diffusion equation (eq 1) is solved with different numbers of velocity corrections (eqs 2 & 3) to understand the contribution of velocity corrections to the collection efficiency. The dimensionless convective diffusion and velocity equations are given in eqs 17–19. Different numbers of velocity corrections in R and/or Y directions were embedded in the mass transport equation and the collection efficiencies evaluated for increasing  up to 16.5.

The results are shown in Figure 5. The PINN solution of the full convective diffusion equation with no correction (*n*_corr, R_ = 0, *n*_corr, Y_ = 0, see eq 20), or up to two corrections in both the R and Y directions (*n*_corr, R_ = 2, *n*_corr, Y_ = 2), was made to investigate the disk fluxes. From Figure 5a, the disk fluxes with different corrections were only slightly different at large , suggesting that more velocity corrections only slightly decreased the disk fluxes. Velocity corrections made more impact on the ring fluxes (Figure 5b) and thus the collection efficiencies (Figure 5c): compared with no correction (black curve), one correction in the R direction (orange curve) increased the ring flux and thus decreased the collection efficiencies, while one correction in the Y direction (light green curve) did the opposite. Ring fluxes and the collection efficiencies were less stable at large  possibly caused by numerical instability when the magnitude of the velocity expanded rapidly with increasing . If one correction in both R and Y directions was applied (purple curve), the effects almost canceled out and the resulting collection efficiencies were 1% ∼ 2% higher than without any correction. Additional corrections beyond the first correction have a minimal effect from the first correction.

**Figure 5:**
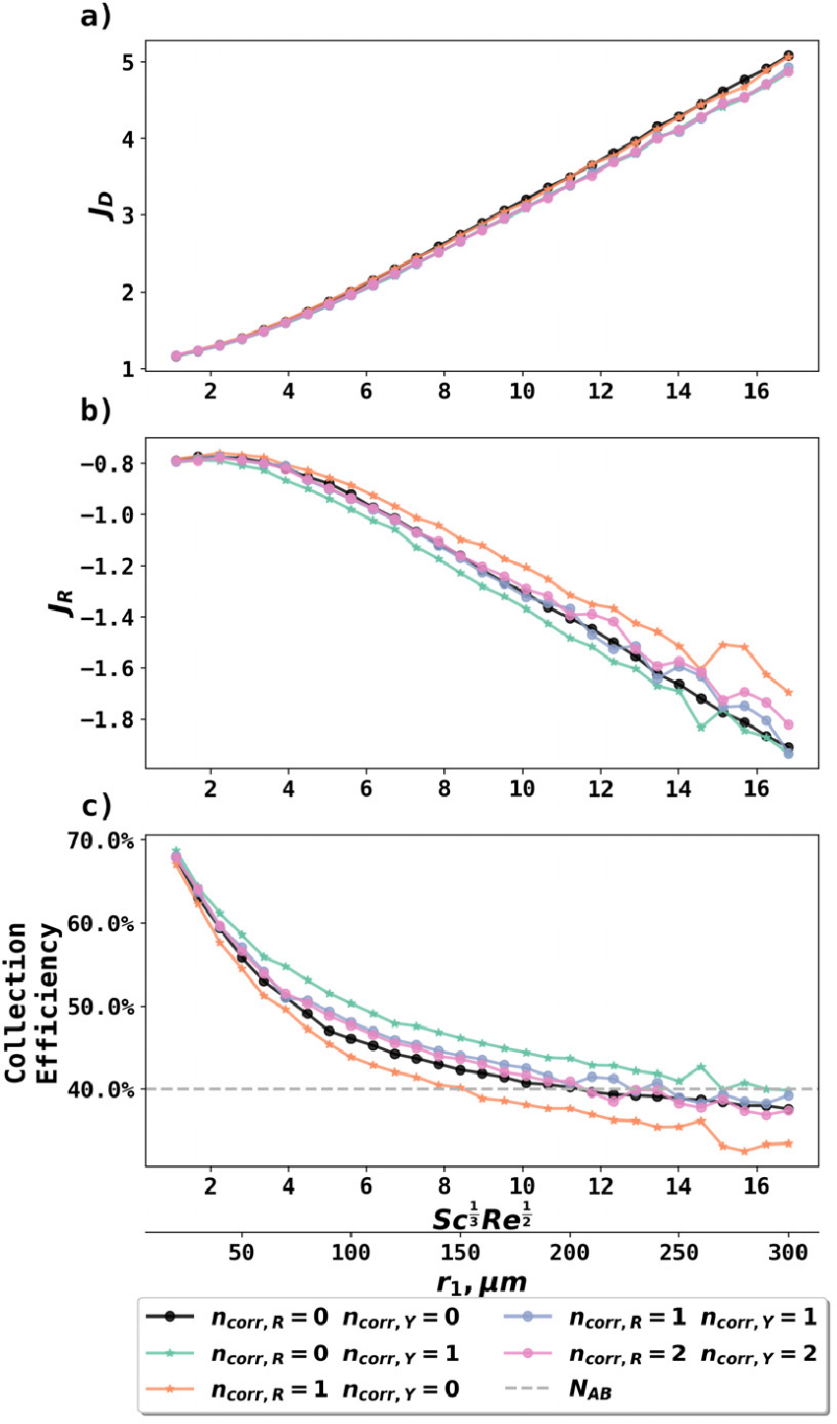
Disk fluxes, ring fluxes, and collection efficiencies predicted by PINN with radial diffusion and different numbers of velocity corrections at increasing .

In summary, PINN was applied to solve the complete convective diffusion mass transport equations with different numbers of velocity corrections and found the importance of velocity corrections to ring fluxes. More accurately solving ring fluxes using PINN will greatly benefit ring transient experiments where absolute ring fluxes are of interest.^31^

The preceding two sections have used model calculations to highlight the extent to which the physical approximations made by Ivanov and Levich, echoed by Bruckenstein and Albery, can limit the accuracy of the derived expressions for the collection efficiency. The discussion of the effects of radial diffusion and edge effects at the RRDE considered a disk radius between 20 to 300 μm while a typical RRDE in practice has a radius of a few millimeters. To help experimental chemists decide when radial diffusion was negligible and thus *N*_AB_ applicable, the disk and ring fluxes shown in Figure 3b illustrate that radial diffusion enhances the fluxes less than 2% if . Figure 6 shows a cutoff plane with three variables: disk radius, rotational frequency, and Sc values, above which the radial diffusion contribution to fluxes was less than 2% such that *N*_AB_ was applicable to within 3%. Figure 6 suggests that *N*_AB_ is approximately applicable at sufficiently larger rotational frequency, radius, and Schmidt number, while PINN may be adopted to explicitly solve the full convective mass transport equation when the experimental parameters were below the cutoff plane. In this way, one can obtain an estimate of the collection efficiency.

**Figure 6:**
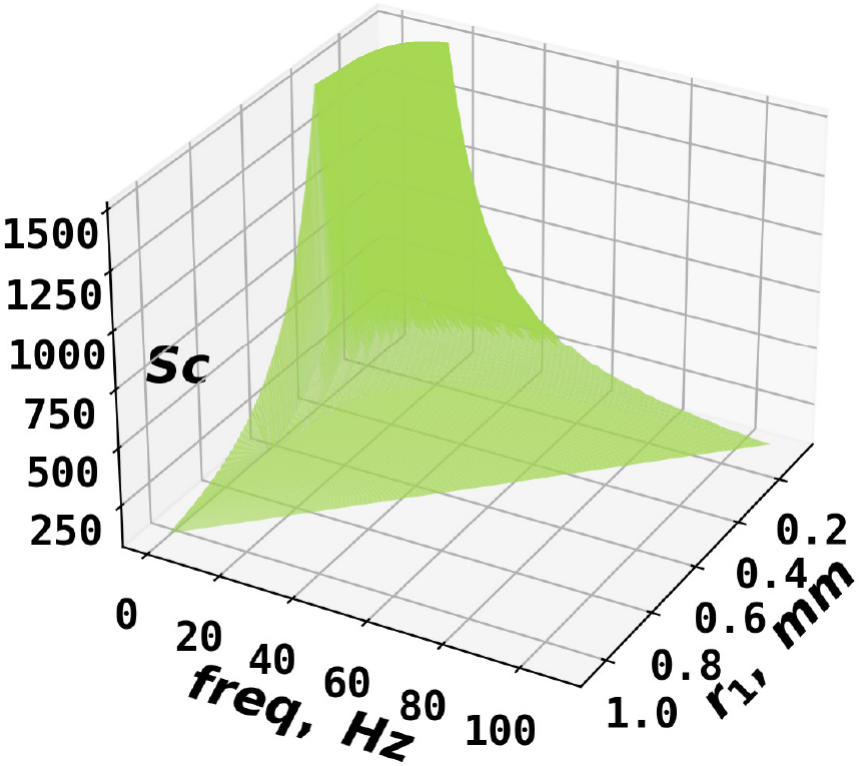
A cutoff plane as a function of disk radius, rotational frequency, and Schmidt numbers above which radial diffusion could be neglected and *N*_AB_ applied.

Importantly, however, the results of Figure 5 show that for RRDEs of practical geometry the radial velocity needs to be corrected beyond the approximations used in the original theories, in addition to the previously explored corrections to the normal velocity noted first for the RDE.^14,15^ The substantial, but partial, cancelation of the effects of extra convective terms on the two velocity components may have encouraged confidence in the physical reality of the approximate model based on eq 20, but the limitations of which become clear under the scrutiny of PINNs. Accordingly, we recommend that if quantitative predictions of collections efficiencies are required then PINN is used to solve the full problem, including multiterm velocity profile equations, while using the Albery/Bruckenstein equation to first give an approximate initial estimate.

65 years after the introduction of RRDE, we have used PINN to explicitly solve the mass transport to the RRDE without the approximations of uniform accessibility, the neglect of edge/radial diffusion effects, or the use of overly approximate velocity profiles. This has led to a better clarification of the physics of the problem and highlighted, particularly, the need to include realistic velocity profiles as well as identifying the role of radial diffusion of ring products recycling back to the disk in enhancing currents at the latter. Using PINN, direct and explicit solutions to the convective diffusion equation with fuller velocity profiles were performed without excessive effort on discretization, suggesting an implementation advantage over conventional finite difference/finite element methods. Most generally, we believe that artificial intelligence, including neural networks, can bring new physical insight into electrochemistry as it enters a new era.

## Experimental Section

The PINN simulation programs were written with Python 3.10, using TensorFlow 2.11. Simulations were performed on clusters with 16 CPU cores and an A100 acceleration card. PINN was trained for 300 epochs, and each epoch took 100–130 s. A PyTorch 2.0 implementation will also be provided at 10.5281/zenodo.11396577.

## Supporting Information Available

Hyperparameter sensitivity test and implementation of convective diffusion equation on neural networks (PDF)
