# Supplementary material for: Removing 65 Years of Approximation in Rotating Ring Disk Electrode Theory with Physics-Informed Neural Networks
Source: J Phys Chem Lett. 2024 Jun 10;15(24):6315–24. doi: 10.1021/acs.jpclett.4c01258 (PMC11194821; doi:10.1021/acs.jpclett.4c01258)
Supplement: Supplementary file 1 — jz4c01258_si_001.pdf [file jz4c01258_si_001.pdf]

Supplementary Information for "Removing 65 Years of Approximation in Rotating Ring Disk Electrode Theory with Physics-Informed Neural Networks"

Haotian Chen[1], Bedřich Smetana[2], Vlastimil Novák[2], Yuanmin Zhang,[1] Stanislav V. Sokolov[3], Enno Kätelhön[4], Zhiyao Luo[5], Mingcheng Zhu[6], and Richard G. Compton[1,*]

[1] Department of Chemistry, Physical and Theoretical Chemistry Laboratory, University of Oxford, South Parks Road, Oxford OX1 3QZ, Great Britain

[2] Department of Chemistry and Physico-chemical processes, Faculty of Materials Science and Technology, VSB - Technical University of Ostrava, 17. listopadu 2172/15, 708 00 Ostrava-Poruba, Czech Republic

[3] St John's College, University of Oxford, St Giles', Oxford OX1 3JP, Great Britain

[4] Offenbach am Main 63067, Germany

[5] Department of Engineering Science, University of Oxford, Parks Road, Oxford OX1 3PJ, United Kingdom

[6] Department of Computing, Imperial College London, Exhibition Road, London SW7 2AZ, United Kingdom

[*] Corresponding Author

Email address: Richard.compton@chem.ox.ac.uk (R. G. Compton)

Table of Contents

Hyperparameter Sensitivity 1

Imposing the full convective diffusion equation onto neural networks; the TensorFlow Implementation .. 2

Hyperparameter Sensitivity

In this section, the sensitivity of hyperparameters was tested. R_0 is a hyperparameter used in 2D simulations of Rotating Ring Disk Electrode, which determines the left boundary of simulation where Levich approximation applies. R_0 was tested at 0.01, 0.02, 0.05 and 0.1 and $R_0 = 0.05$ was used to generate the results reported in this paper. The left boundary was expressed as:

$$C_A = \Gamma\left(\frac{1}{3}, \frac{1}{3}\frac{L}{D}y^3\right), R = R_0, Y = [0, Y_{sim}] \tag{1}$$

PINN simulations of the convective diffusion equation with radial diffusion but without Schmidt number corrections were performed to evaluate the sensitivity of R_0. The response of R_0 to the disk flux, J_D, ring flux, J_R, and collection efficiency with increasing dimensionless rotational speed $Sc^{\frac{1}{3}}Re^{\frac{1}{2}}$ is plotted in Figure S1. R_0 had negligible effect on the disk flux as indicated by Figure S1a while R_0 had decreasing

influence on ring fluxes with increasing $Sc^{\frac{1}{3}}Re^{\frac{1}{2}}$. Collection efficiency was almost unaffected by R_0 as shown in Figure S1c, suggesting that $R_0 = 0.05$ was a valid hyperparameter for solving the rotating ring disk electrode mass transport problem with PINN.

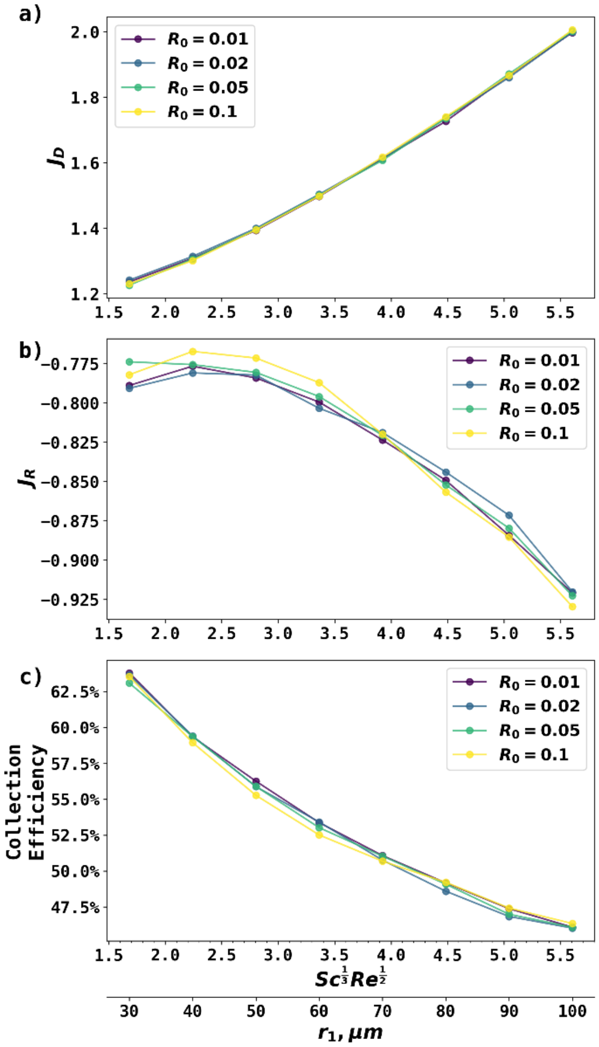

Figure S1. Sensitivity test of R_0 for J_D, J_R and collection efficiency as a function of dimensionless rotational

Imposing the full convective diffusion equation onto neural networks

Simulations of the RRDE requires solution requires solutions to the complete convective-diffusion equation in the form:

$$\underline{V_R}\frac{\partial C_A}{\partial R} + \underline{V_Y}\frac{\partial C_A}{\partial Y} = \frac{\partial^2 C}{\partial Y^2} + \frac{1}{R}\left(\frac{\partial C}{\partial R}\right) + \frac{\partial^2 C}{\partial R^2} \quad (2)$$

where $\underline{V_R}$ and $\underline{V_Y}$ are defined as:

$$\underline{V_R} = \mathcal{L}RY\left(1 - \frac{0.5000}{0.51023}YRe^{\frac{1}{2}} + \frac{0.20533}{0.51023}\ Y^2Re + \cdots\right) \tag{3}$$

$$\underline{V_Y} = -\mathcal{L}Y^2\left(1 - \frac{0.33333}{0.51023}YRe^{\frac{1}{2}} + \frac{0.10265}{0.51023}Y^2Re + \cdots\right) \tag{4}$$

Training the PINN started with large number of collocation points randomly distributed in the R, Y planes as $\{R_i, Y_i\}_{i=1}^{\mathcal{N}}$. Since $\mathcal{L}$ and Re were known parameters for simulations, the velocity profile at each coordinate was computed as the input to the neural network as $\left\{\underline{V_{R,i}}, \underline{V_{Y,i}}\right\}_{i=1}^{\mathcal{N}}$. The inverse of R_i was also computed and used as an input to the neural networks. This preprocessing of data can of course be performed within the neural networks by stopping gradient tracing, although performing this job outside of neural networks can greatly simplify the structure of neural networks and enhance the stability of training for a major time saving. The neural network was denoted as a mapping between the predicted concentration, $\hat{C}_{A,i}$ and the coordinates (R_i, Y_i) as $\hat{C}_{A,i} = \mathcal{f}(R_i, Y_i)$. Conventional data-driven training requires many known concentrations at known coordinates, while physics-informed training does not require that. Instead, physics-informed training minimized the error of the governing equations on all collocation points, which indirectly finds the solution to the equation.

The training workflow of enforcing the convective mass transport equation on the neural network to solve it is visualized in Figure S2,and consisted of four major steps. First, coordinates from the collocation points were passed to a fully connected neural network to predict the concentration at these coordinates. Next, using automatic differentiation feature provided by all major neural network frameworks, the first order and second order partial derivatives were calculated. Third, the partial derivatives, along with velocity profiles and auxiliary variables passed to the neural network as inputs, were used to calculate losses in the form of the governing mass transport equations. Last, the losses were used to optimize the neural network weights. After a few hundred epochs of training, the predicted concentration by the neural network complied with the governing equation and hence the equation was deemed to be solved.

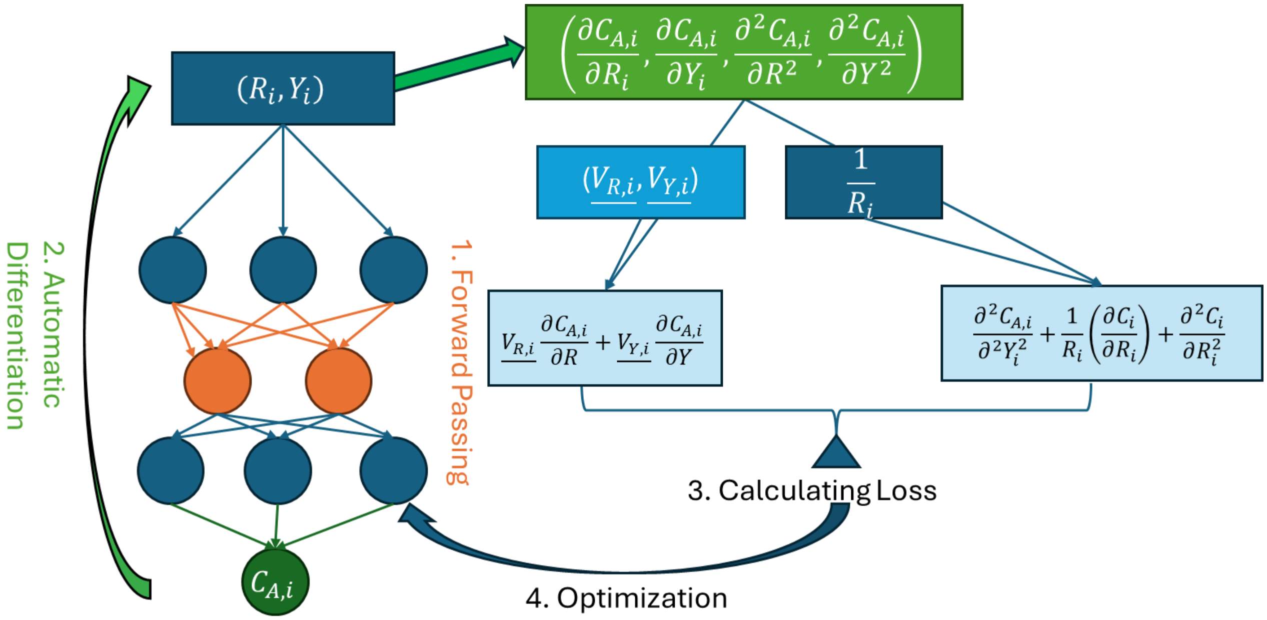

Figure S2. The training scheme of physics-informed neural networks for rotating ring disk electrode by enforcing the convective diffusion mass transport equation on all the collocation points. $R_i, Y_i, \frac{1}{R_i}, \underline{V_R}$ *, and* $\underline{V_Y}$ *are inputs to the neural network.*
